# ADAPT-3D: Accelerated Deep Adaptable Processing of Tissue for 3-Dimensional Fluorescence Tissue Imaging for Research and Clinical Settings

**DOI:** 10.21203/rs.3.rs-6109657/v1

**Published:** 2025-03-24

**Authors:** Daniel D. Lee, Deanna L. Davis, Leon C.D. Smyth, Kevin A. Telfer, Rahul Ravindran, Rafael S. Czepielewski, Christopher G. Huckstep, Siling Du, Kento Kurashima, Ajay K. Jain, Jonathan Kipnis, Bernd H. Zinselmeyer, Gwendalyn J. Randolph

**Affiliations:** Washington University School of Medicine; Washington University School of Medicine; Washington University School of Medicine; Washington University School of Medicine; Washington University School of Medicine; Washington University School of Medicine; Washington University School of Medicine; Washington University School of Medicine; Saint Louis University; Saint Louis University; Washington University School of Medicine; Washington University School of Medicine; Washington University School of Medicine

## Abstract

Light sheet microscopy and preparative clearing methods that improve light penetration in 3D tissues have revolutionized imaging in biomedical research. While most clearing methods focus on removing molecules that scatter light, the methods generally involve immersing tissues in solutions that minimize refraction of light to enhance detection of fluorescent signal deeper into tissues. Here, we developed a new tissue preparative method called ADAPT-3D with broad applicability across species and tissue types. This method enables efficient antibody staining and detection of endogenous fluorophores and offers advantages in terms of speed at which tissue staining and clearing is achieved. In about 4 days from tissue harvest to imaging, human intestinal tissue could be Axed, decolored and delipidated to remove light-interfering substances and stained with antibodies for imaging. In the intact mouse skull and brain, involving an 8-day protocol from tissue harvest to completion of imaging, the aqueous and non-shrinking ADAPT-3D method allowed the specialized channels between skull and underlying tissue to be detected without meningeal tearing. Overall, ADAPT-3D provides a highly versatile preparative method for 3D fixed tissue imaging with superior time savings, sensitivity and preservation of tissue morphology compared with previously described methods.

## Introduction

Over the last decade, interest in 3D fluorescence tissue imaging has flourished, accompanied by the advance of several protocols to refine approaches. While some new imaging modalities like light-sheet microscopy^1^ have contributed to the advances, there have been many refinements in tissue processing to optimize image acquisition using established modalities like tile-scanning confocal microscopy. In 2013, a novel method called CLARITY that aimed to improve light penetration and reduce light diffraction was introduced in which electrical current, urea, and detergents were applied at length to physically remove lipids from organs like mouse brain.^2, 3^ To preserve tissue integrity under these conditions, perfusion fixation and penetration of the tissue with a hydrogel was recommended. While this method worked well with endogenous fluorescent reporters expressed in mouse, antibody penetration through the gel was limited but has improved in further modifications.^4–6^ The introduction of CLARITY coincided with a revived focus on 3D reconstruction of tissue structures, as 3D imaging of long, thin structures like vessels and nerves yields much more informative biology than thin sections.

These reinvigorated efforts in 3D imaging included use of solutions with a refractive index that matches the surrounding tissue to reduce the diffraction of light through the sample and thereby improve light penetration and collection. This practice, referred to as refractive index matching (RIM), was introduced over a century ago when organic mixtures of plant-derived essential oils, methyl salicylate and benzyl benzoate, were applied to fully dehydrated tissue samples that had been decalcified.^7^ Oils like methyl salicylate, for example, have the capacity to match refractive index sufficiently well that light will pass through > 1 millimeter slices of opaque adipose tissue efficiently enough to generate a gross appearance that the fat is as clear to the eye as “glass”^8^, though little to no extraction of fats or molecules occurs.

Novel organic solvent-based techniques called iDISCO (and variants like iDISCO+)^9–11^ and methods based on use of the essential oil ethyl cinnamate^12, 13^ have emerged as powerful ways to achieve RIM from mouse organs to pig brains. Because these methods use organic solvents, they shrink and may distort tissue or denature fluorescent molecules, thereby hindering use in some applications.

Other historical studies demonstrated promising results in RIM were obtained with glycerol.^14^ Over time, the impact of sugars in altering the properties of light scattering became increasingly recognized,^15–17^ supporting an interest in aqueous mixtures for RIM. In contrast to organic solvents, aqueous solutions hold potential to avoid shrinkage of tissues during dehydration and may be less denaturing to fluorescent molecules. Robust RIM can be achieved in an aqueous setting using solutions containing x-ray contrast reagents like iohexol (brand name Nycodenz) or iodixanol, which have a high refractive index (RI, > 1.4). These compounds have a proven safety profile given their use as X-ray or computed tomography imaging contrast reagents such as those found in Ce3D.^18, 19^ Urea is also useful (RI > 1.3), given its hyperhydrating property.^16, 20^ Combinations of X-ray contrast reagents like iohexol and high concentrations of urea allow for faster-acting, aqueous RIM protocols, like CUBIC with the RIM step taking 7 or more days,^20^ Fast 3D Clear with an RIM step of 3 days,^21^ or EZ Clear that achieves RIM of most tissues within 24 h.^22^ Since RIM is only one of a few steps in tissue preparation for imaging, a tissue harvest to imaging time frame is longer for each of these methods than the length of this single step.

Combining RIM protocols with methods that extract lipids and other light-interfering molecules from tissues further improves depth and clarity of 3D imaging. An effective method for delipidation is SHANEL that uses detergents like CHAPS that form small micelles or salt-free amines, rather than detergents like Tween-20 or Triton-100.^9, 18, 21, 23^ Cationic detergents, such as N-alkylimidazole and the cross-linking agent Quadrol (N,N,N’,N’-Tetrakis(2-hydroxypropyl)ethylenediamine) found in aqueous-based CUBIC, efficiently remove pigments like heme from tissue to further facilitate light penetration and reduce background fluorescence.^18, 20^

We took inspiration from these many advances in aqueous-based clearing methods for our 3D tissue imaging applications. In so doing, we also encountered obstacles, like the tendency of urea to crystallize when it was present at high concentrations in clearing solution. To that end, we undertook efforts to further optimize aqueous approaches to achieve rapid, aqueous 3D imaging. We refer to our new solutions and protocols as ADAPT-3D, Accelerated Deep Adaptable Processing of Tissue for 3-Dimensional imaging. Rather than taking days to achieve RIM, our protocol achieves uniform RIM in minutes to hours for most tissues. We couple this RIM step with optimized fixation, decoloring and delipidation. ADAPT-3D minimizes tissue distortion and the possibility of complications like formation of urea crystals. A version of our method includes efficient decalcification for imaging bone attached to soft tissues.

## RESULTS AND DISCUSSION

### Development of ADAPT-3D

Recent literature to develop aqueous, fast-acting clearing, or RIM (refractive index matching), solutions while minimizing toxic chemicals focused on combinations of x-ray contrast solution with urea^21, 22^ Fast action was reported to be 2–3 days for the RIM step alone, not including additional time for staining^21,22^. A solution of 7M urea in 80% iohexol in a method referred to as EZ Clear was especially appealing^22^. However, the preparation of the EZ Clear mixture for RIM is lengthy and requires heating. Furthermore, the solution is subject to crystallization, including after being applied to tissue, due to urea being near its saturation point. We aimed to find an improved solution while working a similar theme of chemicals. Combinations of components were tested to achieve optical clarity in tissues within hours, preservation of signal to noise, retention of endogenous fluorescent tracers, low toxicity, minimal browning from Maillard reactions, a refractive index as high as possible, maintenance of tissue morphology, and ease of preparation and use. We determined that iodixanol could be used as a base solution, allowing us to start at a refractive index of 1.43 and to proceed without the need for heating when other chemicals were added. To counteract tissue swelling that occurred with iodixanol used alone, we paid close attention to the inclusion of sugars that would partially dehydrate tissue and thereby offset the expansion effect of iodixanol. Although we aimed to reduce the high, crystallization-prone urea concentration associated with EZ Clear, we observed that inclusion of some urea accelerated the speed at which the solution permeated the tissue to achieve RIM. Ultimately, a particular combination of sucrose (30% wt/vol), urea (25% wt/vol), iohexol (~ 26% wt/vol, alternatively known as Histodenz) dissolved into commercially prepared iodixanol (alternatively known as OptiPrep) satisfied all desired characteristics outlined above and resulted in refractive index of 1.50–1.51. We named this formulated RIM solution ADAPT-3D (Table). We also formulated three additional solutions for (i) decolorization, that based on its formulation is expected to lead to some delipidation as well; (ii) an additional solution for partial delipidation; and (iii) decalcification (Table), as described in more detail below.

**Figure 1:**
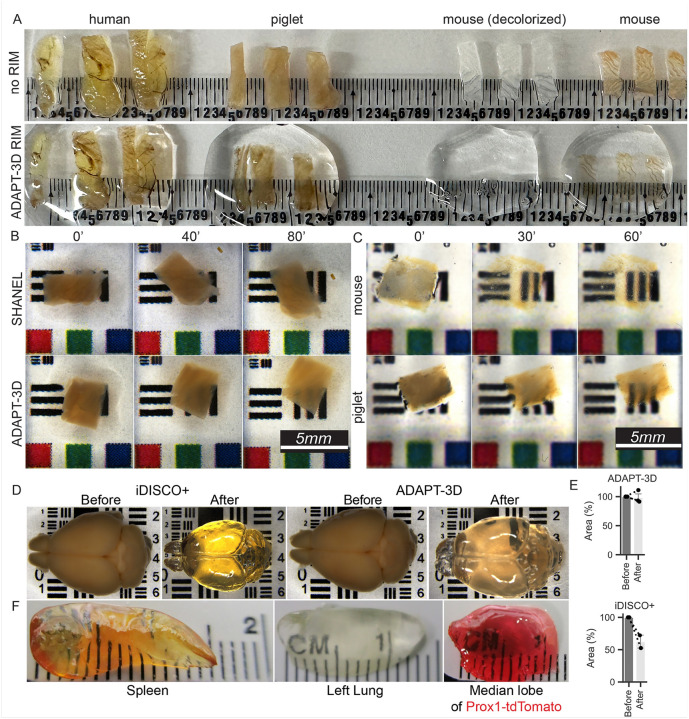
Use of ADAPT-3D on rendering tissues transparent to the eye without shrinkage. A) Illustration of the impact on tissue transparency to the eye after application of ADAPT-3D RIM solution to triplicate samples of fixed human, piglet, and mouse colons for 10 minutes without decolorization or delipidation. In group noted (decolorized), decolorization solution was applied overnight for mouse colon before immersion in ADAPT-3D RIM for 60 minutes. B) Effect of decolorization solution on full-thickness pieces of piglet colon using SHANEL or ADAPT-3D as a function of time over two 40-minute intervals. C) Effect of the ADAPT-3D RIM solution as a function of time illustrated on fixed mouse or piglet colon samples following decolorization and delipidation for 60 minutes, respectively. D) Before and after top-down view of fixed whole mouse brains by stereoscope following iDISCO+ method and RIM in ethyl cinnamate for 4 hours or application of ADAPT-3D decolorization for 48 hours days, delipidation for 36 hours, and RIM solutions for 4 hours. E) Area of the brain surface images measured in duplicate samples after application of the iDISCO+ method or ADAPT-3D decolorization, delipidation, and RIM solutions as mentioned in D. Symbols on the graph depict paired samples before or after. F) Demonstration of mouse spleen or lung clearing using ADAPT-3D, with decolorization for 48 hours and delipidation for 36 hours, with one liver lobe sample derived from Prox1ERCre × TdTomato^fl/fl^ mouse that was decolorized for 72 hours and delipidated for 36 hours followed by RIM incubation overnight.

To test the utility of the ADAPT-3D solution for RIM, we evaluated whether use of the solution alone could render full-thickness samples of human, piglet, and mouse colon transparent. Some mouse colon samples were first decolored using a previously described buffer containing CHAPS and N-methyldiethanolamine^23^ before the samples were exposed to ADAPT-3D RIM solution. Within 10 minutes at room temperature, the newly formulated RIM solution rendered the mouse tissue see-through by eye (Fig. 1A, far right). However, without decoloring and delipidation steps, pigment from heme and lipids that remained in the tissue (Fig. 1A, far right) prevented reaching full transparency in human, piglet, and to a certain extent, mouse intestine (Fig. 1A).

For removal of pigment, we adapted the previously published decoloring solution^23^ by adding limited amounts of N-butyldiethanolamine to increase tissue permeability while preserving morphology and 1,2 hexanediol, an inert emulsifying and humidifying agent (Table) to the solution of CHAPS and N-methyldiethanolamine to generate a faster-acting decolorant. Reagents like hexanediol are used in skin and hair care products and thus we deemed them potentially safe. As for the other solutions we used in ADAPT-3D, we consulted *in silico* resources including those from previous chemical screening to generate the CUBIC-L protocol^18^, Reaxys, Open Reaction Database, and WolframAlpha for potential unwanted reactivity between chemicals and found none. Consistent with this conclusion, we noted that the preparation of this solution did not generate a notable exothermic or endothermic reaction. Decolorization alone (without RIM solution) rendered piglet ileum partially transparent by eye over an 80-minute timeframe (Fig. 1B), appearing more somewhat more efficacious in the same timeframe than the earlier formulated SHANEL decolorant^23^ (Fig. 1B).

To promote partial removal of light-interfering lipids, we aimed to develop a protocol that, while removing tissue lipids, would not lead to shrinkage that is caused by some solutions, such as graded exposure to tetrahydrofuran^21^ that has been long recognized for its delipidation properties.^9, 24, 25^ Although shrinkage can be reversed by rehydration^21^, any initial tissue shrinkage can cause irreversible structural tears in tissue. We considered that hydrating reagents like 1,2-hexanediol were also useful in delipidation^26^. We observed that a balanced mixture of tetrahydrofuran and 1,2 hexanediol, using the mixture delineated in the Table, delipidated tissues well without shrinkage (Fig. 1C–E). Consecutive use of solutions for decoloration/delipidation (80 min), partial delipidation (2 h), and then the ADAPT-3D RIM solution (1 h) (Table) rendered full-thickness piglet colon visibly transparent (Fig. 1C, lower row of images). Thin tissues like mouse colon responded more quickly and more completely in this 3 h trial (Fig. 1C, upper row of images). Fixed, intact brain from a perfused mouse could be rendered visibly transparent in 4 h using ADAPT-3D without shrinkage following 1 overnight incubation of decolorization and 2 days of delipidation, for a total of 4 days of processing post-fixation. In contrast, the popular iDISCO + method cleared a whole, intact brain from a perfused mouse was also processed in 4 days after fixation but was accompanied by significant shrinkage (Fig. 1D) that we quantified (Fig. 1E), consistent with previous work where shrinkage accompanying iDISCO + clearing led to tearing of the mouse dural membrane from skull.^27^ We also successfully cleared other mouse organs, including the heme-rich mouse spleen or the mouse lung (Fig. 1F, left and middle panels), including liver from a mouse with endogenously fluorescent hepatocytes and lymphatic vessels (Prox1^CreER^ × TdTomato^fl/fl^ reporter mice) such that the tissue retained red intensity from the tomato reporter (Fig. 1F, right panel). In summary, our newly formulated ADAPT-3D protocol consisting of three solutions that decolor, delipidate, and match refractive index (Table) collectively supports rapid tissue processing for 3D imaging while avoiding tissue shrinkage at all stages and reducing the time of decolorization, delipidation, and RIM, such that even relatively thick tissues, like the 1.5 mm pig colon wall, could be effectively prepared for imaging in under 5 h. Overall, ADAPT-3D is simple to prepare, store, and use at room temperature, which are features that highlight strong potential for broad adaptability to fluorescence imaging.

### Light-sheeting imaging with ADAPT-3D including at the intact skull-meningeal interface

**Figure 2:**
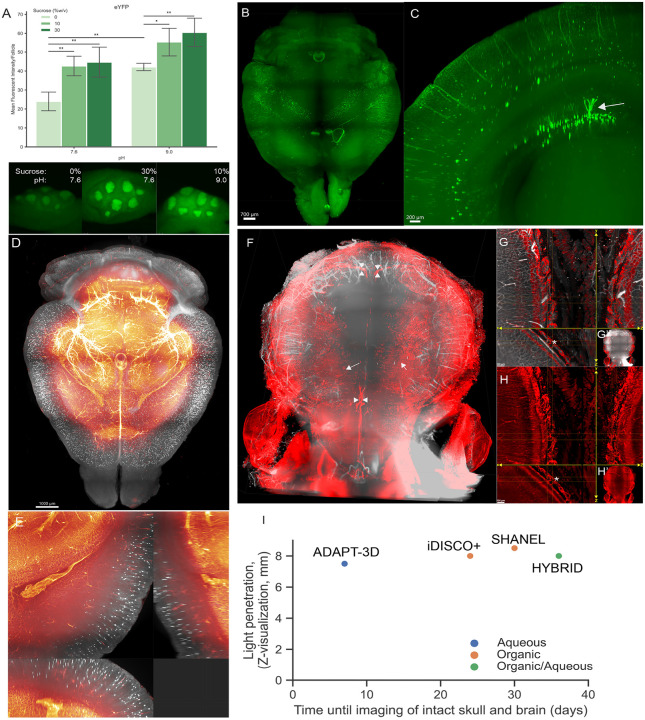
Effect of ADAPT-3D on retaining intensities of endogenous fluorescent reporter proteins and visualization across skull-brain interface. A) Comparison of fluorescent intensities of CD11c-eYFP follicles in Peyer’s patches of mouse ileum following different fixative conditions (*n* = 4–5 follicles per condition, two-way ANOVA followed by Tukey test, *p-value ≤ 0.05, **p-value ≤ 0.01) and captured by stereomicroscopy. B) 3D whole-mount projection of a brain from 16 weeks old mouse expressing CD11c-eYFP which was decolorized, delipidated, applied in refractive index matching solution followed by imaging with light sheet microscopy. C) 500-micron coronal projection from whole brain of CD11c-eYFP from (B). White arrow points to a CD11c-positive neuron. D) 3D whole-mount projection of a brain from ChAT-CreER × TdTomato^fl/fl^ mouse injected retro-orbitally with Lectin-Dylight649 to label vasculature. E) Extended display near ventricle from d displaying preservation of fine dendrites from neurons. F) Whole mount projection of Lyve1CreER × TdTomato^fl/fl^ mouse injected with i.v. with Lectin-Dylight649 and CD31-Alexa Fluor647. G and H) Extended display displaying skull channels. Asterisk depicts consecutive skull channels emanating across skull to leptomeninges. I) A graphical depiction of the relationship between depth of light penetration in intact mouse brains and skull imaged using light-sheet microscopy compared with the reported times in the literature for other protocols listed on the graph or the 7-day time frame determined here for ADAPT-3D.

We wondered if ADAPT-3D could be utilized in light-sheet imaging. Before moving forward, we optimized tissue fixation to retain intensity of sensitive endogenous fluorescent reporters while limiting masking of antigens that would be detected by immunolabeling. Based on prior studies using recombinant fluorescent reporter proteins (FP), we investigated how altering pH, temperature, and sugars might augment the intensity of the FPs^28–30^. Systematic surveys of different fixation conditions were performed that utilized 4% w/v paraformaldehyde (PFA) as the base fixative. We measured the intensity of lymphoid follicles containing CD11c-eYFP^+^ cells within Peyer’s Patches of mice (Fig. 2A). The lowest average CD11c-eYFP signal intensity was observed in the most commonly used fixative, PFA at neutral pH 7.6 (Fig. 2A). As is well known, the addition of the known protein stabilizing agent sucrose at least 10% (w/v) further improved retention of fluorescence intensity (Fig. 2A). Adjustment to pH 9.0 alone provided a modest retention of signal intensity. Addition of at least 10% (w/v) sucrose to 4% (w/v) PFA at pH 9.0 was as efficient for fluorescence intensity of eYFP at 30% sucrose at pH 7.6 (Fig. 2A). For some tissues, however, fixation with 30% sucrose was sufficiently dehydrating as to cause tissue compression.

Illustrating the rapid transparency afforded by ADAPT-3D and its capacity to be coupled with light-sheet imaging of endogenous fluorophore reporter proteins like the sensitive fluorescent protein eYFP,^31, 32^ we applied the ADAPT-3D protocol using 2 days of decolorization and 2 days of delipidation followed by RIM for 4 hours to the imaging of brains from CD11c-eYFP transgenic mice^33^ or ChAT-TdTomato^fl/fl^ reporter mice that identify cholinergic neurons^34^ using light-sheet microscopy. Mice were injected i.v. with Dylight649-labeled Lycopersicon Esculentum lectin to label the vasculature and the fixed brains were treated with ADAPT-3D solutions in succession over a total of 4 days before light-sheet microscopy (Fig. 2B–E). In some CD11c-EYFP^+^ transgenic mice, we unexpectedly identified EYFP^+^ cells with morphology of dentate gyrus granule cells (Fig. 2B, C). This finding likely points to an off-target expression pattern in a mouse strain designed to study antigen-presenting dendritic cells of the immune system, but nonetheless nicely highlights the depth and preservation of eYFP that ADAPT-3D offers in whole mouse brain light-sheet imaging. In ChAT-TdTomato^fl/fl^ reporter mice injected i.v. with Dylight649-labeled lectin for vascular contrast, the fine dendrites of ChAT neurons in the cortex and vessels throughout the brain were resolved (Fig. 2D, E; Video 1). Thus, in 4 days from tissue collection to imaging, ADAPT-3D methodology cleared the whole mouse brain for light-sheet microscopy with strong retention of the fluorescent intensities of endogenous reporter proteins eYFP and tdTomato.

A challenge for 3D imaging is the examination of bone with adjacent soft tissues such as in the skull-brain interface where reported skull channels exist through which immune cells and vasculature are observed^35–37^. Previous studies highlight this challenge with use of popular methods like iDISCO, where the organic nature of the iDISCO solutions cause dehydration and consequent shrinking of soft tissue that tear the fragile space leptomeninges between the brain and skull, while simultaneously extinguishing endogenous fluorophores.^27^ As a result, visualization of the leptomeningeal space including its skull channels has been limited. We decalcified the fixed intact brain and skull using EDTA with N-butyldiethanolamine, imidazole, and 1,2-hexanediol at pH 9.0 (Table) over a period of 3 days at room temperature while preserving endogenous fluorophores. In LYVE1^CreER^ × TdTomato^fl/fl^ mice injected i.v. with Lectin-Dylight649, we observed through the intact skull meningeal macrophages (Fig. 2F, arrows) and dural lymphatic vessels (Fig. 2F, arrowheads) through the intact skull. In fact, there were distinctive LYVE1^+^ channels bridging the skull and the leptomeningeal space, some of which were Lectin^+^ and some that were not (Fig. 2G, H, asterisks; Video 2). The time from tissue acquisition and the initiation of processing to the time of light-sheet imaging with an intact skull was 8 days (1 day fixation, 2 days decolorization, 1 day delipidation, 2 days RIM, such that light-sheet imaging was set up on the 6th day). ADAPT-3D compared favorably with depth of clearing of brains or brain slices with an intact skull, as it was significantly faster from start to finish than all reported methods investigating light-sheet brain imaging including iDISCO,^27^ SHANEL,^5^ or HYBRID.^5^ A comparison of total time frame used in ADAPT-3D relative to these other published processes is charted in Fig. 2I. Among other aqueous-based methods, CUBIC and its variants rendered marmoset brain hemispheres transparent in 29 days without attached skull.^38^

### Preserving fickle antigens with deep immunolabeling

**Figure 3:**
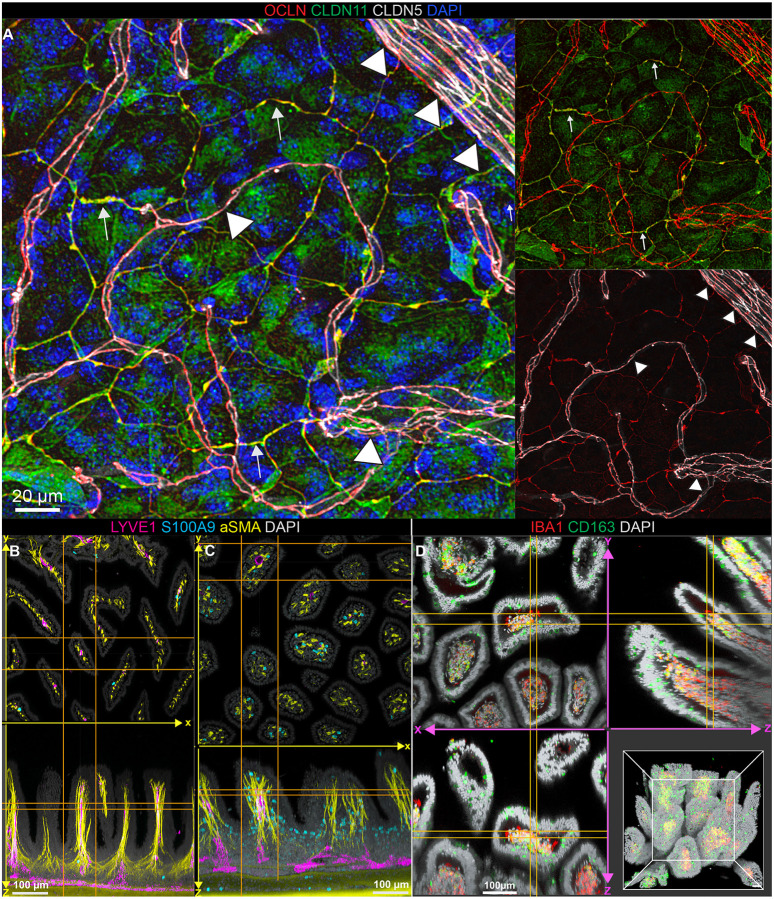
Effect of ADAPT-3D on finicky antigens and compatibility with deep immunolabeling. A) Maximum intensity projections of tight junctions (arachnoid barrier: occludin in red and claudin-11 in green, endothelial-cell specific: claudin-5 in grey) found in leptomeninges from mouse imaged by confocal microscopy. B) Extended display showing en face and z-side projections of mouse ileum that was immunolabeled with alpha smooth muscle actin (yellow), lymphatic vasculature (LYVE-1, magenta), myeloid cells (S100A9, cyan), and nuclei (DAPI, grey) followed by imaging with confocal microscopy. C) Extended display showing en face and z-side projections of ileum from a 16-week-old mouse that expresses Tnf^ΔARE^, a model of ileitis. D) Extended display showing en face, z-side, and 3D projections of fixed human ileum applied with decolorization, delipidation, immunolabeling with CD163 (green), IBA1 (red), and nuclei (DAPI, grey) followed by refractive index matching.

Having observed that fluorescent reporter proteins are preserved with strong intensity, we next asked how immunolabeling was affected in the ADAPT-3D protocol. In particular, some antigens such as tight junctions are commonly masked with traditional fixation using 4% PFA at neutral pH, which have led some to seek alternative fixatives including those using methanol^39, 40^. Our modified fixative (Fig. 2A) combined with ADAPT-3D clearing enabled detection of claudin-11 and occludin tight junctional proteins along the arachnoid barrier and claudin-5- and occludin-expressing endothelial cells in the leptomeninges (Fig. 3A). Use of the modified methanol fixative in a side-by-side comparison revealed our approach was superior (Supplemental Fig. 1).

As a further illustration of the utility of ADAPT-3D with immunolabeling protocols, we imaged full thickness preparations of the mouse ileum, staining for smooth muscle actin (SMA) and S100A9 in ileum of wildtype littermates (Fig. 3B, Video 3) and TNF^ΔARE^ mice (Fig. 3C, Video 4) that develop transmural ileitis^41^. Widened, edematous villi and infiltrated neutrophils associated with ileitis in TNF^ΔARE^ mice were evident (Fig. 3B, C; Videos 3, 4). ADAPT-3D was also used to visualize macrophage subpopulations differentially expressing CD163 and IBA1 in villi of the human intestine (Fig. 3D, Video 5). Villus height was readily measured, here averaging 344.6 ± 38.4 ^m mean ± S.E.M.). Video 5 depicting these images in 3D reconstruction adds staining with the dye hydrazide. Macrophages with the capacity to take up hydrazide dye were described in the mouse colon as cells guarding against toxin exposure^42^ In the human ileum, a subpopulation of hydrazide-stained IBA1^+^ macrophages was apparent, localizing in the lower depths of the villi (Video 5).

In summary, we show that ADAPT-3D is a superior method for processing tissue, decalcifying bone and achieving RIM that holds numerous advantages for 3D tissue imaging. It is remarkably fast-acting compared with the reported tissue processing times of other aqueous methods. The use of compounds in a mixture and ratio that readily penetrates tissue likely underlies its fast action and ease of use. As we illustrate, ADAPT-3D is also designed to prevent morphological changes that result from dehydration, and is readily applicable toward optimal preservation of fluorescent reporters without the need to add compounds to “boost” these reporters. It also readily accommodates antibody staining. Finally, it holds advantage in the area of low toxicity. Recently, dichloromethane, a component in iDISCO and other related methods, was regulated by the environmental protection agency such that laboratory exposure will require monitoring (40 Code of Federal Regulations Part 751). ADAPT-3D does not use this regulated chemical, adding another attractive characteristic that should favor its adaptability for imaging applications in light microscopy. We suggest that this protocol will be of strong interest to pathologists and scientists interested in 3D fluorescence imaging.

## Methods

### Mouse tissue

All mice were bred and housed in specific pathogen-free facilities at Washington University School of Medicine under standard housing conditions (12-hour light and dark cycles with feeding *ad libitum)*. The Institutional Animal Care and Use Committee (IACUC) approved all experiments and procedures (protocol 22–0433 to GJR). All animal experiments were conducted in accordance with the relevant guidelines. This study was conducted in accordance with the ARRIVE guidelines (Home | ARRIVE Guidelines). Apart from CD11c-eYFP and CD11c-eYFP × Prox1CreER-tdTomato^fl/fl^ mice, all other mice were on a C57BL/6 background. CD11c-EYFP mice^33^ were a gift from Michel Nussenzweig (Rockefeller University). Prox1^CreER^ mice^43^ were from Jackson Laboratory (Jax# 022075) and crossed to tdTomato^fl/fl^ mice^44^ from Jackson Laboratory (Jax #007909). ChAT-TdTomato^fl/fl^ reporter mice (Jax #028861), originating from a cross between were a gift from the Rodney Newberry laboratory at Washington University. For tdTomato expression in inducible Cre mice, 12-week-old CD11c-eYFP × Prox1CreER-tdTomato^fl/fl^ mice were treated with 2 mg tamoxifen (Sigma Aldrich, T5648), given by gavage and dissolved in sterile corn oil to 20 mg/mL for 3 total doses within a 7-day period. TNF^ΔARE/+^ mice^41^ were obtained in 2016 through the Cleveland Digestive Disease Research Core Center (NIH P30 DK097948) and continuously bred at Washington University. These mice were kept and bred as heterozygotes and always cohoused with wild-type littermates. LYVE^CreER^ tdTomato^fl/fl^ mice were generated by the Kipnis laboratory as described^45^, and administered 2 mg tamoxifen by oral gavage 3 times over 1 week before euthanasia to process samples for imaging.

### General ADAPT-3D preparation and immunostaining

The following is a generalizable workflow for the processing of samples. However, the specific durations and volumes varied depending on the tissue type and size. In general, following fixation in modified ADAPT-3D fixative at 4°C ranging from 4 hours to overnight, all steps are performed at room temperature. Samples were rinsed twice in 1X phospho-buffered saline (PBS) containing 10 U/mL heparin with at least 5 times of excess volume of the tissue. If bones are included, samples were immersed in excess volume of Decalcification Buffer (Table) at room temperature with daily change until soft to the touch. Hours after incubation with Decolorization/Delipidation Buffer (Table), samples become visibly partially transparent while incubation is generally performed for 12 hours every 1 mm of tissue; they were then washed in 1X PBS containing 10 U/mL heparin where visible transparency appears to reverse, which generally took about 1 hour at room temperature. Samples were incubated in Partial Delipidation Buffer (Table) until some transparency (especially apparent for the brain) was observed followed by washing in 0.2X PBS for at least 1 hour at room temperature until exchanged for 1X PBS containing 10 U/mL heparin. Finally, if just visualizing fluorescent reporter proteins, samples are immersed in Refractive Index Matching Solution until transparent. If immunolabeling was planned, samples were incubated in ADAPT-3D blocking buffer (containing 0.1 mM Glycine, 0.167% Tween-20, 0.33% Triton X-100, 1% Donkey Serum, 1% Alpaca Serum, 1% BSA, 0.05% Hydrogen peroxide, 5% v/v DMSO) with antibodies (see protocol below) rinsed with 1X PBS containing 10 U/mL heparin and 0.2% Tween-20, and then refractive index matching solution (Table) until transparent. For most tissues, they were acclimatized in 0.5X ADAPT-3D Refractive Index Matching solution diluted in 1X PBS for 30 minutes to 1 hour at room temperature before exchanging into 1X ADAPT-3D Refractive Index Matching solution until transparent.

### Mouse tissue immunostaining and imaging

For tight junctional protein staining, following euthanasia, C57BL/6J mice were perfused by transcardiac perfusion with PBS containing 10 U/mL heparin then by modified ADAPT-3D fixative. For preparation of mouse intestines, samples were fixed overnight in modified ADAPT-3D fixative, rinsed twice with 1X PBS containing 10 U/mL Heparin for 30 minutes each, incubated in Decolorization/Delipidation Buffer for at least 60 minutes, incubated overnight with antibodies against alpha smooth muscle actin-Cy3 (clone 1A4, 1:200, Sigma-Aldrich, C6198), Lyve1 (Abcam, ab14917, 1:300), S100A9 (Bio-techne R&D, AF2065, 1:400) in ADAPT-3D blocking buffer. Samples were rinsed in 1X PBS containing 10 U/mL and 0.2% (v/v) Tween-20 for 30 minutes at room temperature. They were then mounted in 0.5X ADAPT-3D Refractive Index Matching solution in 1X PBS for 30 minutes at room temperature before incubating in 1X ADAPT-3D Refractive Index Matching solution until transparent. For comparisons of preserving tight junctions in leptomeninges, samples were fixed with either a 4-h incubation with 100% methanol (wt/vol) or left in ADAPT-3D fixative. The following pre-treatments of ADAPT-3D methodology as described above were performed including decolorization and delipidation. For immunolabeling, dorsal cortices were roughly dissected with a razor blade (0.5–1 mm thick) and stained with antibodies against Occludin (clone OC-3F10, Invitrogen, Cat. No., 331594, 1:200), Claudin-5 (clone 4C3C2, Invitrogen, Cat. No., 352588, 1:100), Claudin-11 (Invitrogen, Cat. No., 36–4500, 1:100) in ADAPT-3D blocking buffer. Samples were rinsed in 1X PBS containing 10 U/mL and 0.2% (v/v) Tween-20 at room temperature. Finally, cortices were mounted in 0.8 mm CoverWellTM imaging chambers containing refractive index matching solution.

### Piglet tissue

Experiments involving piglets were approved by Institutional Animal Care and Use Committee (IACUC) protocol 2346 at Saint Louis University to AKJ and registration 43-R-001 to AKJ from the United States Department of Agriculture (USDA). All experiments were conducted in accordance with relevant guidelines. Piglets were bred at and obtained from Oak Hill Genetics (Ewing, IL). Piglets were procured at 7 days of age and then housed for 14 days before experimental study and euthanasia. During this period, they were fed with Nutra-Start Liqui-Wean formula (Milk Specialties Global, Eden Prairie, MN). Euthanasia was performed in compliance with the approved protocol through an overdose injection of sodium pentobarbital. Tissue was then immediately collected and transferred to fixative in large buckets to allow for nearly 5 times the volume of tissue. Following fixation, tissues were washed with 1X PBS containing 0.3M glycine for 1 hour at room temperature, for a total of 3 changes. They were then stored at 4°C until proceeding with the protocol.

### Human tissue preparation and immunostaining

Human intestinal tissue samples were collected according to and in compliance with IRB protocols # 201111078 (to Rodney Newberry on behalf of the Digestive Diseases Research Core Center at Washington University) and #201111038 (to GJR) approved by the Washington University Human Research Protection Office / Institutional Review Board (HRPO / IRB). **All experiments were performed in accordance with the relevant guidelines.** Informed consent to acquire tissue from surgical or pathological waste was collected from each participant using protocol #2001111078. The research reported here was associated with tissue obtained under protocol # 201111038 that, as per these approved protocols, collects tissues procured under protocol # 201111078, using a waiver of consent. Accordingly, and in compliance with the associated regulations, no protected health information or identifiers were collected. No tissues were acquired from prisoners, fetuses, or pregnant women. For some intestinal samples, tissue was perfused with fixative directly into the ileocolic artery of the mesentery followed by immersion in fixative overnight at 4° C. 1 cm^3^ samples were rinsed in 1X PBS containing 10 U/mL heparin for 2 hours, incubated in decolorization/delipidation buffer for 2 days, partial delipidation buffer for 1 day, followed by blocking buffer containing antibodies against CD163 (EDHu-1, Bio-Rad, 1:100), IBA1 (Fujifilm Wako, 019–19741, 1:100), and DAPI (Sigma-Aldrich, D9542, 1:200) for 2 days. Samples were rinsed in 1X PBS containing 10 U/mL Heparin and 0.2% Tween-20 for 2 hours and then incubated in refractive index matching solution until imaging.

### Stereomicroscopy and confocal microscopy and tissue processing

Stereomicroscopy was performed using a Leica M205FA stereoscope equipped with a K8 digital color camera (2048 × 2048 pixels) at 12-bit for the acquisition of CD11c-eYFP-positive signal. Widefield microscopy was performed using a Zeiss Z1 examiner with an Objective LD SC Plan-Apochromat 20x/1.0 Corr M32 85mm equipped with a Colibri 7 light source. Confocal microscopy was performed with an inverted Leica SP8 microscope that is equipped with 7 lasers with full spectral hybrid detectors (20X Objective lens, NA0.75, oil immersions, or 25X Objective lens, NA0.95, water immersion). Alternatively, high-magnification images were acquired with the Stellaris TCS SP8 confocal microscope (Leica) using either a 10x objective (NA 0.4, Leica) with 2–2.5X digital zoom.

For comparison of visual transparency in organs like brain, spleen, and lung, stereomicroscopy was used. Following euthanasia with CO_2_, 12-week-old C57BL/6J mice were perfused by transcardiac administration with 1X PBS containing 10 U/mL heparin (PBS-H) followed by either 4% PFA (pH 9.0) for ADAPT-3D or 4% PFA (pH 7.6) for iDISCO^+^ brains and post-fixed overnight at 4°C. For ADAPT-3D processing of the lung and spleen, following fixation, they were washed in PBS-H for 30 minutes at room temperature and then incubated in ADAPT-3D Decolorization buffer for 48 hours. After washing in PBS-H for 30 minutes at room temperature, they were then incubated in ADAPT-3D delipidation buffer for 24 hours, washed in PBS-H again for 30 minutes at room temperature, and then incubated in RIM solution for 4 hours.

For clearing using iDISCO^+^ methodology, brains were processed according to the protocol available at http://idisco.info with the only exception that the refractive index matching solution was ethyl cinnamate. Briefly, after overnight fixation, brains were dehydrated in graded methanol solutions starting at 20% v/v in MilliQ water for 1 hour at room temperature, progressing to 100% methanol, followed by 1 hour in chilled 100% methanol. The brains were delipidated with a 2:1 dichloromethane:methanol solution at room temperature and washed in 100% methanol two times and chilled at 4°C. Then, they were incubated overnight in chilled fresh 5% hydrogen peroxide in methanol at 4°C, rehydrated in a decreasing methanol series at room temperature until they were twice washed in 1X PBS containing 0.2% Triton X-100 for 1 hour at room temperature. Brains were again dehydrated in a progressive series of methanol into a 2:1 dichloromethane:methanol solution when they were incubated for 3 hours at room temperature, twice washed in 100% dichloromethane for 15 minutes, then incubated in ethyl cinnamate for 4 hours. Following iDISCO^+^ or ADAPT-3D processing, samples were imaged with Leica M205 stereoscope that is mounted with DFC7000T camera. Areas were quantified by outlining brains at fixation or after refractive index matching.

### Light-sheet microscopy and processing

Lyve1-CreER mice were injected retro-orbitally with a mixture of 40 micrograms of Lectin-Dylight649. After 5 minutes, mice were euthanized. Intact skull with brains were fixed overnight at 4°C, twice washed in 1X PBS containing 10 U/mL heparin (PBS-H) for 30 minutes each at room temperature, incubated in ADAPT-3D decalcification buffer with daily change for 3 days, washed in PBS-H once for 30 minutes at room temperature, and incubated in ADAPT-3D Decolorization buffer for approximately 48 hours at room temperature. They were washed in PBS-H once for 30 minutes at room temperature followed by incubation in ADAPT-3D delipidation buffer for approximately 36 hours, washed in PBS-H twice for 30 minutes at room temperature. Finally, they were incubated in 0.5X RIM in PBS to acclimatize the tissue for 1 hour at room temperature and then 1X RIM overnight.

For intact brains from CD11c-eYFP and ChAT-tdTomato reporter mice, following retro-orbital injection, the steps above were performed with the exception of decalcification. Following incubation in ADAPT-3D refractive index solution, samples were then placed in immersion oil (Cargille Labs, NA 1.52). Images were acquired on Miltenyi Ultra Microscope Blaze with a 4X/NA0.35 using 633 nm at 13% power with 40 millisecond exposure, 568 nm at 11% power with 20 millisecond exposure. Following acquisition, ome-tiff file formats were used to stitch in Stitchy^™^ (Translucence Biosystems) with default settings and exported as *.ims.

### Image visualization

3D visualization was visualized on Imaris software (Bitplane Inc.) on v10.1.1 software.

### Statistical analysis

Data are presented as arithmetic mean ± standard deviation. Two-way ANOVA was performed to test the null hypothesis that there is no difference between mean intensities between pH or sucrose amounts followed by Tukey post-hoc test. p-values ≤ 0.05 were considered statistically significant. Experiments were repeated at least three times.

## Figures and Tables

**TABLE.** ADAPT3D formulation

ADAPT-3D Refractive Index Matching Solution		
*Reagent*	*Amount*	*Final Concentration*
Iohexol	7.6 g	25.33% (w/v)
D-Sucrose	9.0 g	30% (w/v)
Urea	7.5 g	25% (w/v)
n-propyl gallate	0.150 g	0.5% (w/v)
1-thioglycerol	0.3 mL	1% (v/v)
Iodixanol	To 30 mL	
ADAPT-3D Decolorization/Delipidation Buffer		
*Reagent*	*Amount*	*Final Concentration*
CHAPS, ((3-cholamidopropyl)dimethylammonio)-1-propanesulfonate	100 g	10%
N-methyldiethanolamine	250 mL	25%
N-butyldiethanolamine	50 mL	5%
1,2-hexanediol	100 mL	10%
Water	To 1L	
ADAPT-3D Partial Delipidation Buffer		
*Reagent*	*Amount*	*Final Concentration*
Tetrahydrofuran	5 mL	50%
1,2-hexanediol	5 mL	10%
ADAPT-3D Decalcification Buffer		
*Reagent*	*Amount*	*Final Concentration*
EDTA	150 g	15% (w/v)
Imidazole	150 g	15% (w/v)
N-methyldiethanolamine	100 mL	10% (v/v)
N-Butyldiethanolamine	50 mL	5% (v/v)
Water	To 1L	

## Data Availability

Primary data are present within the manuscript. Raw image files in LIFF format can be made available upon request to GJR, BHZ, or DDL.
